# Supplementary material for: Ultrafast all-optical second harmonic wavefront shaping
Source: Nat Commun. 2024 Mar 20;15:2507. doi: 10.1038/s41467-024-46642-9 (PMC10954747; doi:10.1038/s41467-024-46642-9)
Supplement: Supplementary file 1 — Supplementary Information [file 41467_2024_46642_MOESM1_ESM.pdf]

Supplementary Information

Ultrafast all-optical second harmonic wavefront shaping

Artem Sinelnik[1,2], Shiu Hei Lam[2], Filippo Coviello[1,2,3], Sebastian Klimmer[1], Giuseppe Della Valle[3,4], Duk-Yong Choi[5], Thomas Pertsch[2,6,7], Giancarlo Soavi[1], Isabelle Staude[1,2,7*]

[1]*Institute of Solid State Physics, Friedrich Schiller University Jena, 07743 Jena, Germany*

[2]*Abbe Center of Photonics, Institute of Applied Physics, Friedrich Schiller University Jena, 07745 Jena, Germany*

[3]*Dipartimento di Fisica, Politecnico di Milano, Piazza Leonardo da Vinci, 32, 20133 Milano, Italy*

[4]*Istituto di Fotonica e Nanotecnologie, Consiglio Nazionale delle Ricerche, Piazza Leonardo da Vinci, 32, 20133 Milano, Italy*

[5]*Laser Physics Centre, Research School of Physics, Australian National University, Canberra ACT 2601, Australia*

[6]*Fraunhofer Institute for Applied Optics and Precision Engineering, 07745 Jena, Germany*

[7]*Max Planck School of Photonics, 07745 Jena, Germany*

isabelle.staude@uni-jena.de

These authors contributed equally: Artem Sinelnik, Shiu Hei Lam, Filippo Coviello

*Corresponding author isabelle.staude@uni-jena.de

Design principle of the metasurfaces

Three metasurfaces were designed to realize different ultrafast-switching functions, namely Gaussian-to-vortex-beam switching, topological charge switching and beam deflection. The first metasurface switches between vortex beam and non-vortex beam, the second switches between vortex beams with +1 and -1 topological charge, while the last metasurface switches the beam steering from a positive angle to a negative angle. The required phase profiles, $\phi_{\mathrm{LCP}}(x,y)$ and $\phi_{\mathrm{RCP}}(x,y)$, in the two operating modes of the three metasurfaces respectively are summarized in Supplementary Table 1. The modulation of phase of light by the particles on the metasurface is based on the principle of Pancharatnam-Berry phase, to be explained in this section. For the metasurface for Gaussian-to-vortex beam switching, in which an addition degree of freedom for phase modulation is required, the manipulation of propagation phase was also employed in the design.

Consider an elliptic-cylindrical meta-atom with its long- and short- axis aligned with the x- and y-axis, the Jones matrix of the meta-atom reads

$$\widehat{T_0} = \begin{pmatrix} T_{xx} & 0 \\ 0 & T_{yy} \end{pmatrix}.$$

A rotation of the meta-atom along the z-axis with an angle θ would result in the modified Jones matrix of the meta-atom:

$$\hat{T}(\theta) = \hat{R}(-\theta)\widehat{T_0}\hat{R}(\theta) = \begin{pmatrix} T_{xx}\cos^2\theta + T_{yy}\sin^2\theta & (T_{xx} - T_{yy})\cos\theta\sin\theta \\ (T_{xx} - T_{yy})\cos\theta\sin\theta & T_{xx}\sin^2\theta + T_{yy}\cos^2\theta \end{pmatrix},$$

where $\hat{R}(\theta) = \begin{pmatrix} \cos\theta & \sin\theta \\ -\sin\theta & \cos\theta \end{pmatrix}$ is the rotation matrix. An input LCP/RCP light $E_{\pm} = (1 \pm i)$ would result in an output light with two parts:

$$E_{\pm}^{\mathrm{out}} = \hat{t}(\theta)E_{\pm} = 1/2(T_{xx} + T_{yy})E_{\pm} + 1/2(T_{xx} - T_{yy})e^{\pm i2\theta}E_{\mp},$$

where the subscript in $E_{\pm}^{\mathrm{out}}$ indicates the input polarization.

The two parts are the co-polarized output, of which the polarization is the same as the input, and the cross-polarized output, of which the polarization is orthogonal to the input. The phase of the cross-polarized output scales linearly with the geometric rotation angle θ of the meta-atom, while the direction of scaling depends on the handedness of the input light. Employing properly designed meta-atom which suppress the co-polarized output would allow the manipulation of phase of output light through the geometric phase 2θ.

The geometric phase provides one degree of freedom for the spatial control of phase as the input light propagates through the metasurface. This one degree of freedom is sufficient for constructing a polarization-dependent metasurface in which the phase modulation profile $\phi(x,y)$ of one polarization

is the negative of the other, i.e. $\phi_{\text{LCP}}(x,y) = -\phi_{\text{RCP}}(x,y)$. The topological charge switching and the beam steering metasurface can hence be solely based on the principle of geometric phase. The metasurface for Gaussian-to-vortex beam switching, however, requires a spatial profile different from that allowed by merely geometric phase. In such cases, the propagation phase which light acquires during the propagation in the meta-atom is also exploited. The geometric phase controlled by the rotation angle of the meta-atom and the propagation phase controlled by the dimension of the meta-atom provide two degrees of freedom and allow an arbitrary phase profile for each polarization input.

The design of the metasurfaces required the search for a suitable set of meta-atoms which suppresses the co-polarized output and allows 2π-propagation phase modulation. The conditions can be written in terms of the Jones matrix components as follows:

1. $|T_{co}|^2 = |1/2(T_{xx} + T_{yy})|^2$ is minimized,
2. $\arg(T_{cross}) = \arg(1/2(T_{xx} - T_{yy}))$ spans the range $[-\pi, \pi)$.

For the need to operate in the non-resonant regime that maximizes transmission, elliptical nanopost was selected to be the meta-atom. Hydrogenated amorphous silicon (a-Si:H) was appointed as the meta-atom material for its availability of fabrication resources and low absorption at the operation wavelength (750 nm). The lattice constant *p* of the meta-atoms array was set at 450 nm such that the metasurface is in the non-diffractive regime, while the inter-particle interaction is negligible.

The Jones matrix components T_{xx} and T_{yy} of a-Si:H nanoposts with different height *h*, major and minor semi-axes, r_x and r_y at wavelength 750 nm were evaluated using the finite difference method in frequency domain with CST Studio Suite. The system was modelled as periodic identical nanoposts on glass substrate with lattice constant *p*. The model of nanopost was built with a side wall angle α of 3.5° to resemble the fabrication limit. The refractive index of nanoposts for the simulation were experimentally measured from a sample of a-Si:H (*n* = 3.77), while the refractive index of the substrate is set to 1.45. A schematic of the nanoposts is provided in Supplementary Fig. 1.

From the calculation result, it is observed that with height *h* = 490 nm it is possible to provide a set of nanoposts, which fulfill our optimization condition. Among all nanoposts with h = 490 nm, the nanoposts with r_x= 45 nm shows minimal co-polarized output, while Arg(T_{cross}) spans approximately full 2π range by varying r_y (Supplementary Fig. 2). Based on the above result, the height of the nanoposts for all metasurfaces were set to 490 nm. Nanoposts with r_x = 45 nm and r_y ranging from 60 nm to 180 nm were included in the metasurface for Gaussian-to-vortex beam switching to fulfill the 2π variation of propagation phase. The topological charge switching metasurface and beam steering metasurface, which do not require the manipulation of the propagation phase of the nanoposts, were

constructed with only one type of a-Si:H nanopost (r_x = 60 nm and r_y = 185 nm), which gives maximal contrast of cross-polarized output to co-polarized output. The 3D models of the nanopost with the selected dimension (r_x = 60 nm, r_y = 185 nm), the all metasurface are accessible on https://doi.org/10.6084/m9.figshare.25112717.v1.

Theoretical calculation of vortex beam propagation through a cylindrical lens

The calculation considers the propagation of the vortex beam through a cylindrical lens to free space with diffraction theory. The computation was performed within the scalar approximation, and the cylindrical lens is treated as a thin element with phase profile $\phi = \frac{2\pi}{\lambda}\left(f - \sqrt{f^2 + l^2}\right)$. l is the coordinate along the axis of the cylindrical lens, i.e. $l = x\cos\theta + y\sin\theta$, where θ is the rotation angle of the cylindrical lens. The focal length of the cylindrical lens, 50 mm, and the distance from the cylindrical lens to the detection plane, 200 mm, were taken from the experimental setting. In order to account for the orientation of the cylindrical lens in the experiment, the cylindrical lens axis is rotated by $\theta = -60$ degrees about the propagation z axis.

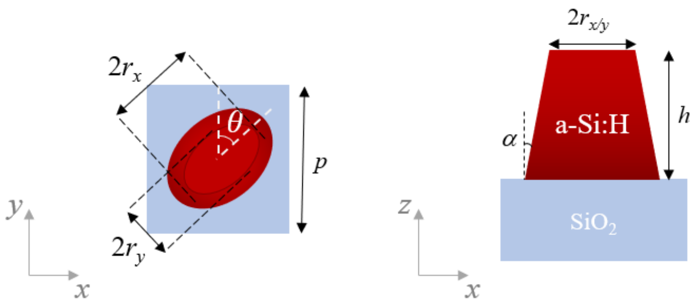

Supplementary Fig. 1. Schematics of the unit cell geometry. The lattice constant p, major and minor semi-axes r_x and r_y, rotation angle $\boldsymbol{\theta}$ and height h of the nanopost, as well as the Cartesian coordinate system are specified.

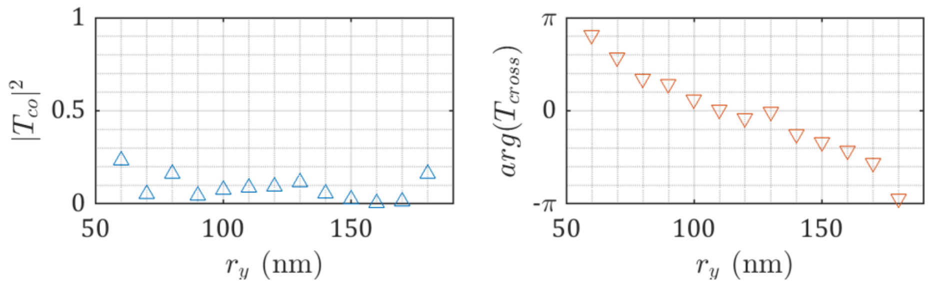

Supplementary Fig. 2. Calculated optical properties of the metasurface in periodic approximation. Transmission $|\boldsymbol{T}_{\text{co}}|^2$ and phase $\arg(\boldsymbol{T}_{\text{cross}})$ of the metasurface design employed for Gaussian-to-vortex beam switching in dependence of the nanopost minor semi-axis r_y. The height h and major semi-axis r_x of the nanoposts are 490 nm and 45 nm, respectively.

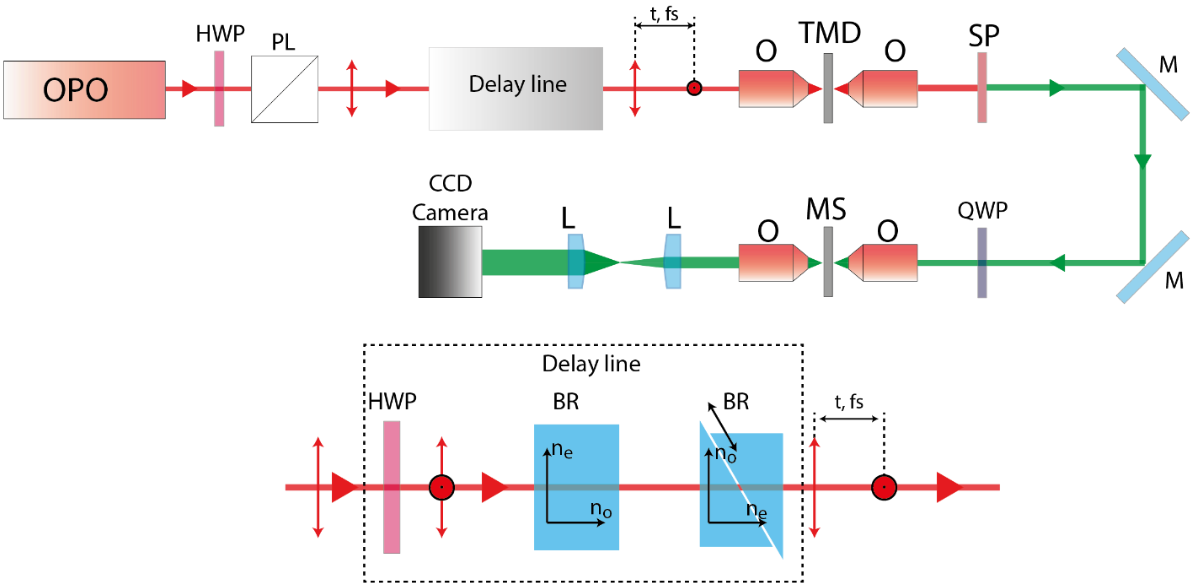

Supplementary Fig. 3. Sketch of the home-built optical setup used for ultrafast second-harmonic wavefront control. The upper panel shows the entire setup, while the bottom panel provides a closeup of the delay line section. Abbreviations: OPO – optical parametric oscillator, HWP – half waveplate, PL – polarizer, O – objective, TMD – transition metal dichalcogenide, SP – short pass filter, M – mirror, QWP – quarter waveplate, MS – metasurface, L – lens, BR – birefringent crystal.

Figure S3 shows a sketch of the pump-probe setup used to observe the ultrafast change in the wavefront (see main text for a detailed description).

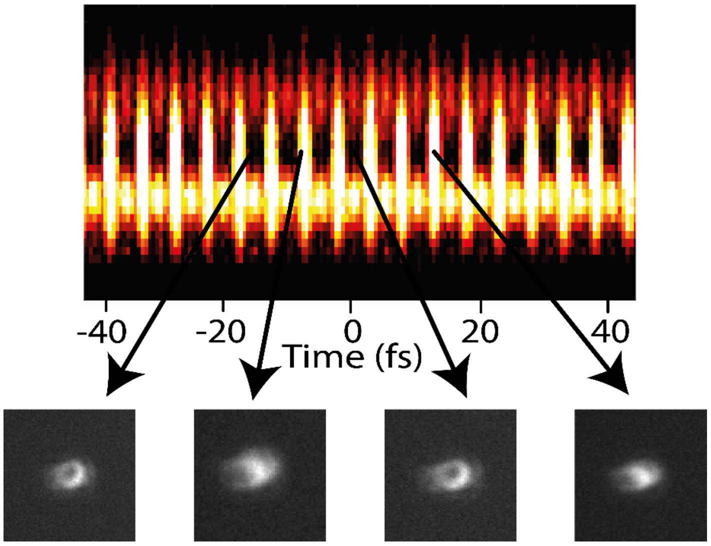

Supplementary Fig. 4. Polarization properties of interferometer output. Fragment of the full interferometric trace (compare inset of Fig. 4 (a)) showing a close-up of the interference fringes. The bottom images show the spatial SH intensity distribution for the delay values marked in the trace. Doughnut shapes are clearly observed for delays corresponding to linear output states of the interferometer.

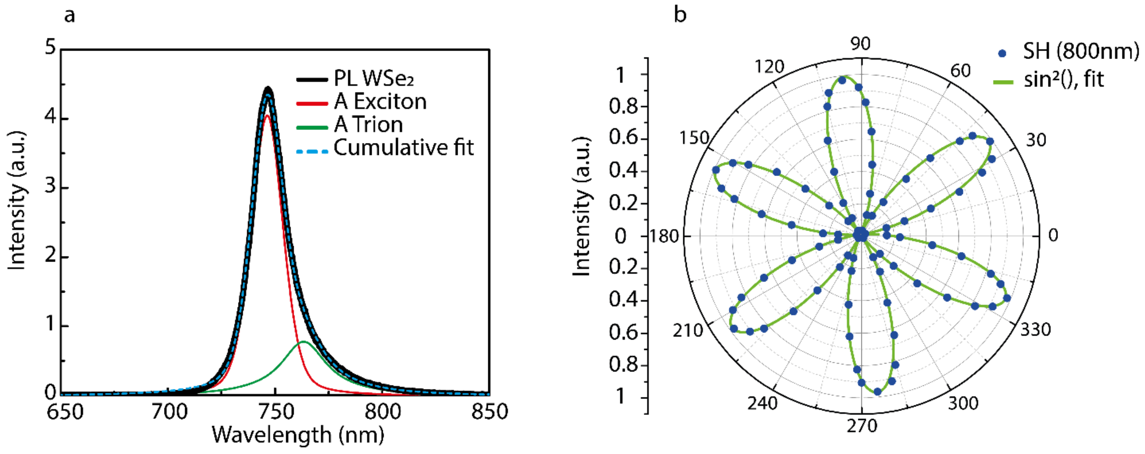

Supplementary Fig. 5. Optical characterization of the WSe_2 monolayer. (a) Photoluminescence (PL) of the WSe_2 monolayer. For the excitation we used a 532 nm CW-laser (Cobolt 08-DPL 532 nm), which was attenuated to an average power of 100 µW and focused onto the sample with an x50 microscope objective (M Plan Apo HL; NA: 0.42). The backscattered PL (black) was subsequently detected with a spectrometer (Horiba, iHR 550 with a Synapse PLUS CCD). Pseudo-Voigt fits show the contributions for exciton (red) and trion (green), respectively. The cumulative fit (dashed blue) agrees very well with the experimental data. (b) Polar plot of the normalized SH intensity a function of the excitation polarization angle δ. The detected SH polarization was always selected parallel to the excitation with a co-rotating wiregrid polarizer (Thorlabs, WP25M-UB) in transmission geometry[1]. As excitation we used the signal output of an OPO (Levante IR from APE) at 1600 nm (10 mW average power; 150 fs pulse duration; 76 MHz repetition rate) which was focused on the monolayer with a x40 reflective objective (Thorlabs, LMM40X-UVV). The blue dots show experimental data and the solid green line indicates the $\sin^2[3(\delta - \delta_0)]$ fit. From the perfectly symmetric six-fold pattern we conclude that no notable strain is present in the investigated sample.

METASURFACE FUNCTIONS	$\phi_{\mathrm{LCP}}(x, y)$	$\phi_{\mathrm{RCP}}(x, y)$
GAUSSIAN-TO-VORTEX BEAM SWITCHING	0	$+\mathrm{Arg}(\boldsymbol{x}+\boldsymbol{iy})/\mathbf{2}$
TOPOLOGICAL CHARGE SWITCHING	$+\mathrm{Arg}(\boldsymbol{x}+\boldsymbol{iy})/\mathbf{2}$	$-\mathrm{Arg}(\boldsymbol{x}+\boldsymbol{iy})/\mathbf{2}$
BEAM DEFLECTION	$+\boldsymbol{ax}$	$-\boldsymbol{ax}$

Supplementary Table 1. A summary of the phase profiles realized for LCP and RCP polarized incident light and for metasurfaces with different functions, respectively.

References

[1] Rosa H. G., et al. Characterization of the second- and third-harmonic optical susceptibilities of atomically thin tungsten diselenide. *Sci. Rep.* **8**, 10035 (2018).
